# Altered Functional Connectivity of the Primary Visual Cortex in Subjects with Amblyopia

**DOI:** 10.1155/2013/612086

**Published:** 2013-06-13

**Authors:** Kun Ding, Yong Liu, Xiaohe Yan, Xiaoming Lin, Tianzi Jiang

**Affiliations:** ^1^State Key Laboratory of Ophthalmology, Zhongshan Ophthalmic Center, Sun Yat-Sen University, Guangzhou 510060, China; ^2^National Laboratory of Pattern Recognition, Institute of Automation, Chinese Academy of Sciences, Beijing 100190, China; ^3^Brainnetome Center, Institute of Automation, Chinese Academy of Sciences, Beijing 100190, China; ^4^Key Laboratory for NeuroInformation of Ministry of Education, School of Life Science and Technology, University of Electronic Science and Technology of China, Chengdu 610054, China; ^5^The Queensland Brain Institute, The University of Queensland, Brisbane, QLD 4072, Australia

## Abstract

Amblyopia, which usually occurs during early childhood and results in poor or blurred vision, is a disorder of the visual system that is characterized by a deficiency in an otherwise physically normal eye or by a deficiency that is out of proportion with the structural or functional abnormalities of the eye. Our previous study demonstrated alterations in the spontaneous activity patterns of some brain regions in individuals with anisometropic amblyopia compared to subjects with normal vision. To date, it remains unknown whether patients with amblyopia show characteristic alterations in the functional connectivity patterns in the visual areas of the brain, particularly the primary visual area. In the present study, we investigated the differences in the functional connectivity of the primary visual area between individuals with amblyopia and normal-sighted subjects using resting functional magnetic resonance imaging. Our findings demonstrated that the cerebellum and the inferior parietal lobule showed altered functional connectivity with the primary visual area in individuals with amblyopia, and this finding provides further evidence for the disruption of the dorsal visual pathway in amblyopic subjects.

## 1. Introduction

Amblyopia is a developmental ocular disorder characterized by a unilateral or bilateral visual deficiency that is out of proportion with any structural abnormalities that are present in the eye [1–4]. It results from neural adaptations to abnormal sensory experiences in childhood. In recent years, extensive neuroimaging studies have found decreased gray/white matter volumes [5–7] and reduced functional activation or connectivity [8–12] in the visual cortical areas or in the visual pathway regions in cases of amblyopia. In a previous study, we also found disrupted spontaneous activity patterns of some brain regions, such as the precuneus, the medial prefrontal cortex, and the cerebellum, in anisometropic amblyopic individuals, which suggested that the decreased visuomotor processing ability and compensatory plasticity coexist in amblyopia [12].

The primary visual cortex (also known as V1, anatomically equivalent to Brodmann area 17 (BA 17)) is a koniocortex (sensory-type cortex) located in and around the calcarine fissure of the occipital lobe. Each hemisphere of the primary visual cortex receives information directly from its ipsilateral lateral geniculate nucleus and transmits information to the dorsal and ventral streams. Previous studies have observed functional deficits and morphological alterations in the lateral geniculate nucleus in cases of amblyopia [13–15], which may suggest that the input pathway could be affected in subjects without normal sight. Yu and colleagues have demonstrated that blind subjects show decreased functional connectivity (functional connectivity may refer to any study examining interregional correlations in neuronal variability [16]. Here, it is a measurement of the spatiotemporal synchrony or correlations of the blood oxygen level-dependent (BOLD) fMRI signal between anatomically distinct brain regions of cerebral cortex.) between the primary visual area and the somatosensory motor areas [17]. Qin et al. [18] suggested that the development of the dorsal and ventral visual areas depends on different visual experiences; these findings support the hypothesis that the development of the human brain is modulated by compensatory plasticity and visual loss effects [12, 19].

The two-stream (dorsal and ventral) hypothesis is an influential and widely accepted model of visual information processing. It is generally believed that the dorsal stream (the “how pathway”), which involves areas such as the middle temporal cortex (MT) and the medial superior temporal area, processes spatial location information. The ventral pathway (the “what pathway”) includes area V4 and the inferior temporal lobe and is associated with the processing of object identification and recognition. Interestingly, numerous psychophysical studies have observed that both the ventral and dorsal extrastriate cortical processing functions are disrupted in amblyopia subjects [20–23]. In particular, the cortical areas extending from V1, including V3a/MT, are implicated in the global motion deficits reported in amblyopia [24–26]. Using an effective connectivity analysis based on task (retinotopic mapping) related functional magnetic resonance imaging (fMRI), Li and colleagues have found that both the feedforward and feedback interactions are anomalous in amblyopia and that this disrupted connectivity extends throughout the thalamocortical pathway [27]. Li and colleagues have also found small but consistent reductions in activation in area V1 when stimulating (spatially broadband) the amblyopic eye compared to that of the fellow fixing eye [28]. However, the functional connectivity pattern of the primary visual cortex in patients with amblyopia remains unclear. The aim of the present study was to investigate the characteristics of the functional connectivity pattern of the primary visual cortex in patients with amblyopia. A group of subjects with amblyopia and their age/gender-matched normal-sighted control subjects were recruited. A correlation analysis was computed between the mean time series of the bilateral primary visual areas and other brain regions. Then, two-sample *t*-tests were accessed in a voxel-wise manner to determine which brain areas showed significant differences between the normal-sighted subjects and the patients with amblyopia for each hemisphere of the primary visual area. 

## 2. Materials and Methods

Parts of the dataset have been used in our previous study to investigate the regional homogeneity of spontaneous activity patterns in amblyopic subjects [12]. To maintain the scientific integrity of the current paper, we also provide a short introduction of the dataset and the preprocessing steps.

### 2.1. Subjects

Written informed consent was obtained from all participants or their legal guardians. This study was approved by the Ethics Committee of Zhong Shan Ophthalmic Center at Sun Yat-sen University and followed the tenets of the Declaration of Helsinki. All participants received detailed eye examinations that included assessments of their visual acuity, intraocular pressure and refraction, slit lamp examination, ophthalmoscopy, binocular alignment, ocular motility, and random-dot butterfly stereograms. In total, fourteen anisometropic amblyopic patients, sixteen mixed (anisometropic and strabismic) amblyopic patients, and twenty-two healthy individuals were enrolled in the study. Three participants (one healthy volunteer and two patients with amblyopia) had excessive head motions during the scanning and were excluded, leaving twenty-one healthy volunteers and twenty-eight patients with amblyopia to be included in the analysis. All of the subjects were right-handed and had no history of other ocular diseases, surgery, neurological disorders, or brain abnormalities based on MRI scans. The volunteers had normal or corrected-to-normal visual acuity in both eyes. Detailed clinical data on the subjects are shown in Table S1 in Supplementary Material available at http://dx.doi.org/10.1155/2013/612086. 

### 2.2. Data Acquisition

The MRI data were obtained using a 3.0 Tesla MR scanner (Trio Tim system; Siemens, Erlangen, Germany). Resting-state fMRI scans were performed with an echo planar imaging sequence with the following scan parameters: repetition time = 2000 ms, echo time = 30 ms, flip angle = 90°, matrix = 64 × 64, field of view = 220 × 220 mm^2^, slice thickness = 3 mm, and slice gap = 1 mm. Each brain volume was composed of 32 axial slices, and each functional run contained 270 volumes. During the scans, all subjects were instructed to keep their eyes closed, relax, and move as little as possible. Tight but comfortable foam padding was used to minimize head motion, and earplugs were used to reduce scanner noise.

The structural magnetization prepared rapid gradient-echo imaging sequence which was used to acquire structural T1-weighted images in a sagittal orientation. The parameters were as follows: repetition time = 2000 ms, echo time = 2.6 ms, flip angle = 9°, acquisition matrix = 512 × 448, and field of view = 256 × 224 mm^2^. The scanning time was approximately 5 min, and a total of 192 images with 1 mm thick slices were obtained.

### 2.3. Data Preprocessing

The fMRI images were conventionally preprocessed using Statistical Parametric Mapping software (SPM8, http://www.fil.ion.ucl.ac.uk/spm/). Detailed preprocessing procedures can be found in our previous study [12].

### 2.4. Region of Interest

The primary visual cortex of the brain generally refers to Brodmann area 17 (BA 17), and the bilateral primary visual cortices were defined using the method used in a previous study [17]. The detailed procedure is as follows: (1) each hemisphere of BA 17 and the gray matter were selected from the TD (Talairach Daemon) Brodmann area atlas; (2) the left BA 17 and the gray matter were intersected to generate the left primary visual cortex; and (3) in the same way, the right primary visual cortex was generated.

### 2.5. Functional Connectivity and Statistical Analyses

Functional connectivity analyses were performed separately for the left and right primary visual cortices. A seed reference time series for each hemisphere of the primary visual cortex was obtained by averaging the fMRI time series of all voxels within the area. A Pearson correlation analysis of the time series was performed between the mean time series and other brain regions in a voxel-wise manner. For further statistical analysis, a Fisher r-to-z transformation was performed to improve the normality of the correlation coefficients.

In this study, we investigated alterations in the connectivity pattern of the visual cortex and other brain areas in amblyopic subjects. A two-sample, two-tailed *t*-test was performed to investigate the group differences in the functional connectivity map of the bilateral primary visual cortex between the anisometropic amblyopic subjects and subjects with normal vision after regressing out the effects of age and gender. The statistical threshold for each voxel was set at *P*_alpha_ < 0.01 with a cluster size of at least 130 voxels based on the results of a Monte Carlo simulation (http://afni.nimh.nih.gov/pub/dist/doc/manual/AlphaSim.pdf; AlphaSim with the following parameters: single voxel *P* = 0.01, FWHM = 6 mm, with the AAL template in the MircroN software as a mask). The same statistical analyses were performed between the mixed amblyopic subjects and normal-sighted subjects and between the anisometropic and mixed amblyopic subjects. Exactly the same statistical analyses were performed for the right primary visual cortex to obtain functional connectivity maps of the right primary visual area.

To evaluate the alterations in the connectivity pattern of the primary visual area in the amblyopic subjects, all of the regions identified from the two comparisons (anisometropic amblyopic subjects versus normal-sighted and mixed amblyopic subjects versus normal-sighted) were overlapped to investigate the impaired regions in the two patient groups. Only regions larger than 70 voxels were identified as significant. 

## 3. Results

The demographic and psychological characteristics of the two amblyopic groups (anisometropic amblyopia: 5 males, 8 females, mean age: 22.3 ± 7.2 years; mixed amblyopia: 8 males, 7 females, mean age: 23.4 ± 7.1  years) are summarized in Table S1. The 21 normal-sighted volunteer individuals (8 males, 13 females; mean age: 23.5 ± 2.1 years) were well matched with the amblyopic group in age (*P* = 0.81, two-sample two-tailed *t*-test) and gender (*P* = 0.616, Chi-squared test). Additionally, an extra evaluation of the differences in movement parameters between subjects with amblyopia and with normal vision was performed according to the procedures described in Van Dijk et al. [29] to further evaluate the influence of head motion on the functional connectivity results. No significant differences were found between the three groups (Table 1).

### 3.1. Altered Functional Connectivity of the Primary Visual Cortex in Subjects with Anisometropic Amblyopia

**Figure 1:**
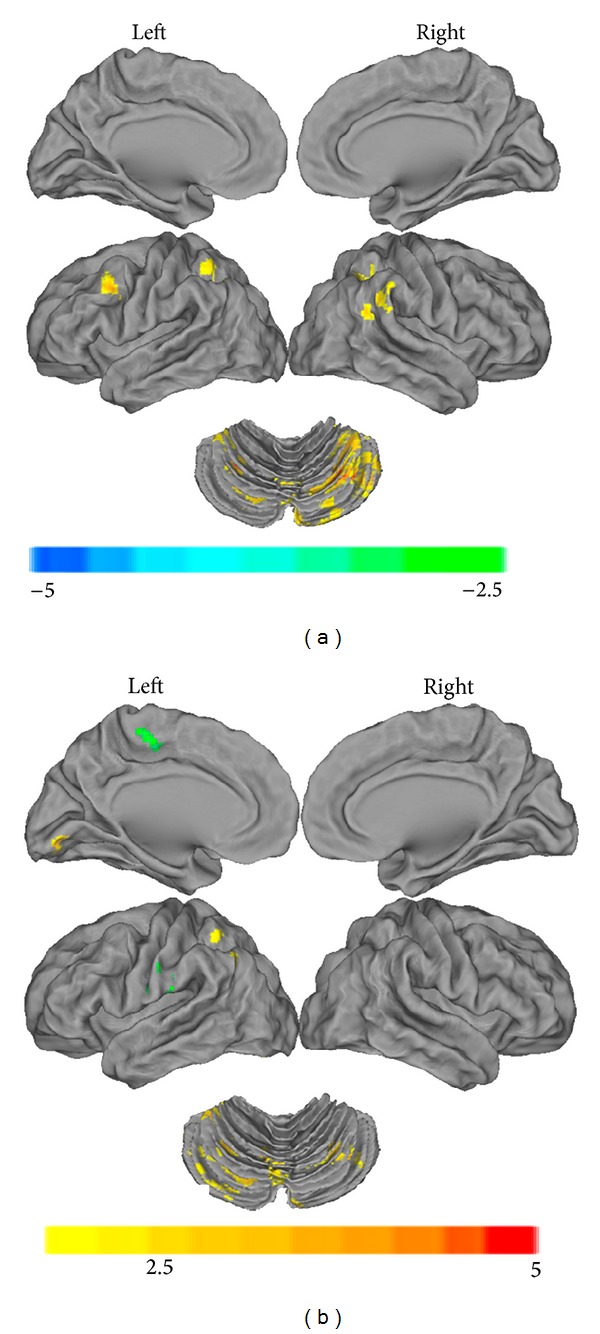
The anatomical distribution of the alterations in functional connectivity with the left primary visual cortex (a) and the right primary visual cortex (b) in anisometropic amblyopia are shown in comparison with normal sighted controls, as individually visualized using the Caret v5.61 software (*P* < 0.01, 130 voxels, AlphaSim corrected *P*_alpha_ = 0.01). A detailed introduction of the brain regions can be found in Tables S2 and S3.

Compared to subjects with normal sight (*N* = 21), anisometropic amblyopic individuals (*N* = 13) showed significantly decreased functional connectivity with the left primary visual area in the cerebellum (left cerebellum 1, right cerebellum crus 1/2, 8/9), the conjunction area of the bilateral inferior parietal lobe and the angular lobe (IPL/ANG, BA 40) and the conjunction area of the left middle frontal lobe and the precentral gyrus (MFG/PreCG.L, BA 8/9) (Figure 1, Table S2). Decreased functional connectivity with the right primary visual area was found in the bilateral cerebellum (left cerebellum crus 1/2 / lingual/vermis_6/9, left cerebellum crus 1/8/9, right cerebellum crus 1/6) and the conjunction area of the left inferior parietal lobe and the angular lobe (IPL/ANG, BA 40), while increased functional connectivity with the right primary visual area was found in the left postcentral gyrus (PostCG.L) and the conjunction area of the left paracentral lobule and the middle frontal gyrus (PCL/MFG, BA 6/31) (Figure 1, Table S3).

### 3.2. Altered Functional Connectivity of the Primary Visual Cortex in Mixed (Anisometropic and Strabismic) Amblyopia

**Figure 2:**
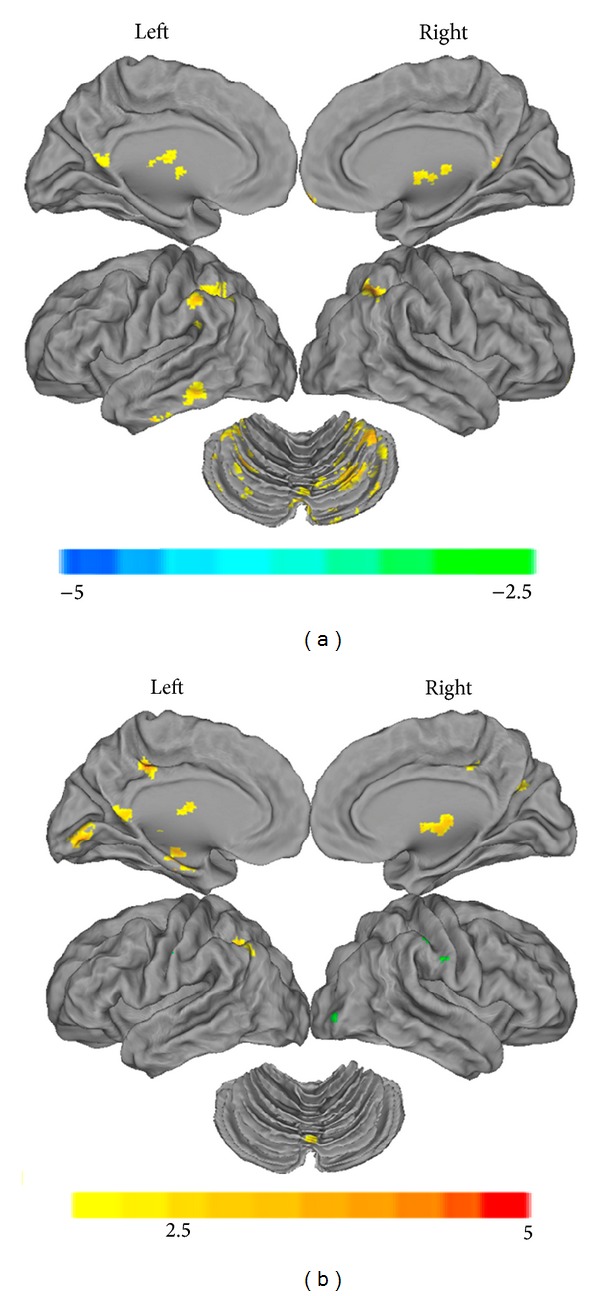
The anatomical distribution of the alterations in functional connectivity with the left primary visual cortex (a) and the right primary visual cortex (b) in mixed amblyopic subjects is shown in comparison with normal sighted controls, as individually visualized using the Caret v5.61 software (*P* < 0.01, 130 voxels, AlphaSim corrected *P*_alpha_ = 0.01). A detailed introduction of the brain regions can be found in Tables S4 and S5.

Compared to the subjects with normal vision (*N* = 21), subjects with mixed amblyopia (*N* = 15) showed significantly decreased functional connectivity with the left primary visual area in the cerebellum (cerebellum crus 1, crus 6/8/9, cerebellum crus6/vermis_9), the conjunction area of the bilateral inferior parietal lobe and the angular lobe (IPL/ANG, BA 7/40), the medial frontal cortex (MFG, BA 11), the conjunction area of the posterior cingulate cortex and the precuneus (PCC/PreCUN, BA 30), the left middle frontal-precentral gyri (MFG/PreCG.L, BA 8/9), the left inferior temporal gyrus (ITG.L, BA 20), and the bilateral thalamus (Figure 2, Table S4). Decreased functional connectivity with the right primary visual area was found in the conjunction area of the left inferior parietal lobe and the angular lobe (IPL/ANG, BA 40), the bilateral conjunction area of the postcentral gyrus and the precentral gyrus (PostCG/PreCG, BA 3/4), the precuneus (BA 31), the conjunction area of the posterior cingulate cortex and the middle cingulate cortex (BA 31), the conjunction area of the left posterior cingulate cortex and the precuneus (PCC/PreCun.L), the lingual gyrus/vermis_6, the middle occipital cortex (MOG, BA 19), and the hippocampus/parahippocampus (HIP/PHIP) (Figure 2, Table S5).

### 3.3. Combined Pathway Impairments of the Primary Visual Cortex in Amblyopia

**Figure 3:**
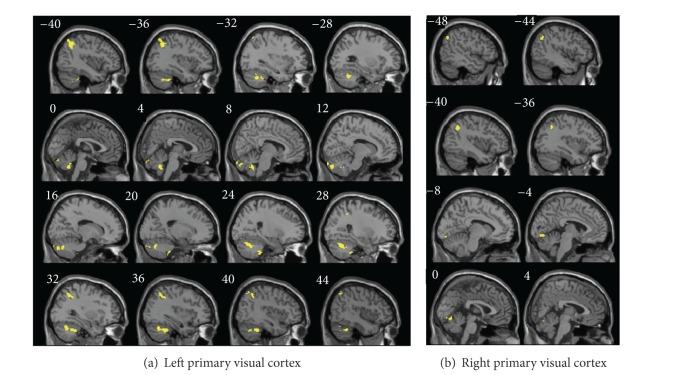
Overlapping brain areas with alterations in functional connectivity with the left primary visual cortex (a) and the right primary visual cortex (b) are shown for amblyopic individuals (cluster size larger than 70 voxels). The details of the regions can be found in Table 2.

We also found overlapping brain areas with altered functional connectivity with the primary visual area in anisometropic and mixed amblyopic individuals (70 voxels). The overlapping brain regions that showed altered functional connectivity with the left primary visual area were located in the cerebellum (cerebellum tonsil, vermis 9/vermis 7, and cerebellum crus 1/6) and the conjunction area of the bilateral inferior parietal lobe and the angular lobe (IPL/ANG) (Table 2). The overlapping brain region showing altered functional connectivity with the right primary visual area was restricted to the adjacent region of the lingual and vermis_6 and the left IPL/ANG (Figure 3, Table 2).

**Figure 4:**
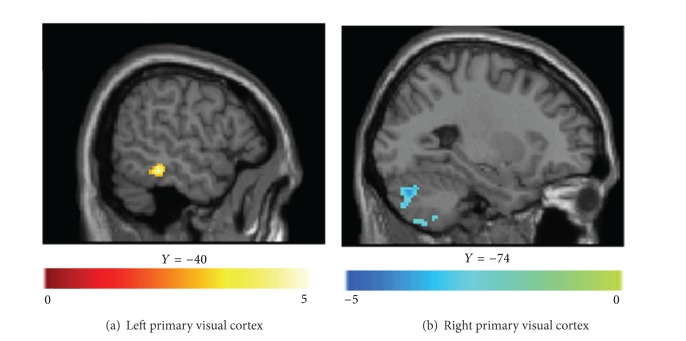
Alterations in functional connectivity with the left primary visual cortex (a) and the right primary visual cortex (b) between anisometropic amblyopic subjects and mixed amblyopic individuals are shown (*P* < 0.01, 130 voxels, AlphaSim corrected, *P*_alpha_ = 0.01). The details of the regions can be found in Table 3.

Compared to the patients with anisometropic amblyopia, patients with mixed amblyopia showed increased functional connectivity between the medial/inferior temporal gyri and the left primary visual area and decreased functional connectivity between cerebellar crus 1/6/8 and the right primary visual area (Figure 4, Table 3).

## 4. Discussion

In the present study, we investigated the functional connectivity between the primary visual cortex and other brain areas in amblyopic individuals using a resting-state functional connectivity technique. From our results, we mainly find significant decreases in functional connectivity with the primary visual area in the inferior parietal lobule and the posterior cerebellum in both anisometropic amblyopia and mixed amblyopia.

The dorsal stream, sometimes called the “where pathway” or the “how pathway”, originates from the V1 area, passes through the V2 and MT (also known as V5) areas, and arrives at the inferior parietal lobule. This pathway primarily participates in the detection of motion, the representation of object locations, and the control of the eyes and arms, especially when visual information is used to guide saccades or reaching behaviors [31, 32]. Recent neurophysiological studies have demonstrated abnormalities in visuomotor processing in subjects with amblyopia [33–36]. The decreased connectivity between the primary visual area and the inferior parietal lobule, which plays a special role in the stereo pathway [37], may also explain the deficit in stereoscopic depth perception observed in subjects with amblyopia. Hence, the decreased functional connectivity between the primary visual area and the inferior parietal lobule in amblyopic individuals may reflect functional deficits in the dorsal stream. In one of our previous studies, Yan et al. [7] found that the dorsal visual pathway was abnormal or impaired in patients with comitant exotropia. The present study provides further evidence for deficits in the dorsal stream in subjects with amblyopia.

We also found a decrease in the functional connectivity between the primary visual area and the cerebellum (cerebellum tonsil, vermis 9, cerebellum crus 2/vermis 7, and cerebellum crus 1/6). The cerebellum, which functionally interacts with the frontal eye fields [38–41], is also involved in the control of eye movements [42–46]. Damage to the cerebellum can affect smooth pursuit eye movement [47]. Thus, we conclude that the observed decrease in functional connectivity between the primary visual area and the cerebellum might explain the visuomotor processing deficits in amblyopia.

In some strabismic subjects, the brain ignores input from the deviated eye. We have found altered functional connectivity between the MTG and the left primary visual cortex and between the cerebellum crus and the right primary cortex in mixed amblyopic subjects compared to anisometropic amblyopic subjects. This might occur because the amblyopic subjects with strabismus would have severely affected gaze judgment and information interaction between the sensory motor and visual areas (Table 3). 

We found increased functional connectivity between the right primary visual area and the left PostCG in cases of anisometropic amblyopia. This corresponds to our previous finding of increased spontaneous activity in the PostCG and PreCG, which may reflect the compensatory plasticity that compensates for amblyopia-related deficits [12]. Nevertheless, it should be noted that we found decreased functional connectivity between the right primary visual area and the conjunction area of the PostCG/PreCG, the thalamus and the hippocampus/parahippocampus in mixed amblyopia (Figure 2). We know that inputs from the retina are sent to the lateral geniculate nucleus of the thalamus, which in turn projects to the primary visual cortex (area V1) in the occipital lobe. Previous studies have also observed functional deficits and morphological changes in the lateral geniculate nucleus in anisometropic amblyopic subjects [13–15] and in some animal studies [48, 49]. The alteration of the functional connectivity between the thalamus and the primary visual cortex might suggest that the lateral geniculate nucleus plays a fundamental part in the processing deficit that has been attributed to the visual cortex in amblyopic subjects. To the best of our knowledge, we did not find a possible reason for the altered functional connectivity between the sensory motor regions and the primary visual cortex; therefore, task-related fMRI studies are needed in the future.

In the initial experimental design of the present study, we only wanted to determine the alteration of spontaneous activity and the functional connectivity pattern in the amblyopic individuals in the resting state. In fact, stereopsis-related changes may provide deeper insight into the neural substrate of the impaired binocular perception in the patient groups. Unfortunately, most of our participants did not have stereopsis scores. Meanwhile, we did not find a statistically significant correlation between altered functional connectivity and disease severity (visual acuity of bilateral eyes) in the patient groups. Furthermore, our results should be interpreted carefully because we did not consider the side of the eye impairments due to the small sample size. In the future, a larger sample neurophysiological and neuroimaging study is required to distinguish the differences among the affected brain regions in the different types of amblyopia.

## Supplementary Material

Table S1. Demographic characteristics of the participants with anisometropia amblyopia.
Table S2. Brain areas alterations in functional connectivity with the left primary visual area between anisometropic amblyopic subjects and normal sighted subjects (P < 0.01, 130 voxels, Alphasim corrected P_alpha_ = 0.01).
Table S3. Brain areas alterations in functional connectivity with the right primary visual area between anisometropic amblyopic subjects and normal sighted subjects (P < 0.01, 130 voxels, Alphasim corrected P_alpha_ = 0.01).
Table S4. Brain areas alterations in functional connectivity with the left primary visual area between mixed amblyopic (anisometropic and strabismic) subjects and normal sighted subjects (P < 0.01, 130 voxels, Alphasim corrected P_alpha_ = 0.01).
Table S5. Brain areas alterations in functional connectivity with right primary visual area between mixed amblyopic (anisometropic and strabismic) subjects and normal sighted subjects (P < 0.01, 130 voxels, Alphasim corrected P_alpha_ = 0.01).

## Figures and Tables

**Table 1:** Demographic, clinical, and neuropsychological data on normal sighted subjects (NC), anisometropic amblyopia (AA) subjects, and mixed amblyopia (MA) subjects.

	NC (*n* = 21)	AA (*n* = 13)	MA (*n* = 15)	*F*-value	*P* value
Gender (M/F)	8/13	5/8	8/7	0.969	0.616
Age (year)	23.5 ± 2.1	22.3 ± 7.2	23.4 ± 7.1	0.211	0.81
Mean head motion	0.51 ± 0.19	0.62 ± 0.33	0.52 ± 0.29	0.794	0.458
Mean rotation	1.48 ± 0.23	1.65 ± 0.26	1.51 ± 0.30	1.868	0.166
Framewise displacement	0.11 ± 0.04	0.13 ± 0.05	0.13 ± 0.08	0.559	0.575

Chi-square analysis was used for gender comparisons, and one-way ANOVA with a Bonferroni post hoc test was used for age and head motion comparisons.

**Table 2:** Overlapping brain areas with altered functional connectivity with the primary visual area in amblyopia individuals (cluster size > 70 voxels).

Brain Region	Cluster Size	MNI Coordinates (*x*, *y*, *z*)
Left primary visual cortex		
		20 −38 −52
Cerebellum Tonsil	85	28 −42 −48
		26 −34 −48
		8 −50 −44
Cerebellum Vermis_9	155	−2 −56 −40
		8 −60 −38
		12 −82 −42
Cerebellum Crus2/Vermis_7	182	18 −82 −36
		6 −84 −34
		−38 −58 −42
Cerebellum_6	178	−38 −48 −40
		−32 −42 −38
		36 −46 −40
Cerebellum Crus1/6	523	16 −72 −38
		40 −64 −38
		−42 −56 36
IPL/ANG.L	269	−36 −60 40
		−44 −60 44
		34 −48 40
IPL/ANG.R	179	40 −54 42
		32 −56 44

Right primary visual cortex		
		4 −74 −16
Lingual/Vermis_6	79	−10 −90 −12
		−2 −70 −12
		−42 −60 34
IPL/ANG.L	117	−48 −66 38
		−36 −56 38

IPL: inferior parietal lobe, ANG: angular lobe, L: left, R: right, MNI Coordinates: Montreal Neurological Institute Coordinates [30].

**Table 3:** Alterations in functional connectivity with the primary visual area between anisometropic amblyopic subjects and mixed amblyopic (anisometropic and strabismic) individuals (*P* < 0.01, 130 voxels, Alphasim corrected *P*_alpha_ = 0.01).

Brain Region	Cluster Size	*T*-scores	*Z*-scores	MNI Coordinates (*x*, *y*, *z*)
Left primary visual cortex				
MTG/ITG	136	5.28	4.26	−52 −36 −18
		3.99	3.46	−62 −44 −18

Right primary visual cortex				
Cerebellum Crus 8	180	−4.52	−3.81	−18 −60 −52
		−3.17	−2.87	−30 −58 −50
		−3.69	−3.25	−26 −74 −32
Cerebellum Crus 1/6	200	3.56	−3.16	−22 −80 −42
		3.37	−3.02	−16 −68 −22

ITG: inferior temporal guys, MTG: middle temporal guys, L: left, R: right, MNI: Montreal Neurological Institute.
