# Supplementary material for: Altered Functional Connectivity of the Primary Visual Cortex in Subjects with Amblyopia
Source: Neural Plast. 2013 Jun 13;2013:612086. doi: 10.1155/2013/612086 (PMC3697400; doi:10.1155/2013/612086)
Supplement: Supplementary file 1 — Table S1. Demographic characteristics of the participants with anisometropia amblyopia. Table S2. Brain areas alterations in functional connectivity with the left primary visual area between anisometropic amblyopic subjects and normal sighted subjects (P < 0.01, 130 voxels, Alphasim corrected Palpha = 0.01). Table S3. Brain areas alterations in functional connectivity with the right primary visual area between anisometropic amblyopic subjects and normal sighted subjects (P < 0.01, 130 voxels, Alphasim corrected Palpha = 0.01). Table S4. Brain areas alterations in functional connectivity with the left primary visual area between mixed amblyopic (anisometropic and strabismic) subjects and normal sighted subjects (P < 0.01, 130 voxels, Alphasim corrected Palpha = 0.01). Table S5. Brain areas alterations in functional connectivity with right primary visual area between mixed amblyopic (anisometropic and strabismic) subjects and normal sighted subjects (P < 0.01, 130 voxels, Alphasim corrected Palpha = 0.01). [file 612086.f1.doc]

**Altered functional connectivity of the primary visual cortex in subjects with amblyopia**

Kun Ding ^1^, Yong Liu ^2, 3, CA^, Xiaohe Yan^1^, Xiaoming Lin^1,CA^, Tianzi Jiang^2, 3, 4,5^

^1^State Key Laboratory of Ophthalmology, Zhongshan Ophthalmic Center, Sun Yat-sen University, Guangzhou, 510060, China

^2^National Laboratory of Pattern Recognition, Institute of Automation, Chinese Academy of Sciences, Beijing, 100190, China

^3^Brainnetome Center, Institute of Automation, Chinese Academy of Sciences, Beijing, 100190, China

^4^ Key Laboratory for NeuroInformation of Ministry of Education, School of Life Science and Technology, University of Electronic Science and Technology of China, Chengdu, 610054, China

^5^ The Queensland Brain Institute, the University of Queensland, Brisbane, QLD 4072, Australia

**Correspondence to**

Dr. Yong Liu

Brainnetome Center &National Laboratory of Pattern Recognition, Institute of Automation, Chinese Academy of Sciences, Beijing, 100190, China

Email: [yliu@nlpr.ia.ac.cn](mailto:yliu@nlpr.ia.ac.cn)

Dr. Xiaoming Lin

State Key Laboratory of Ophthalmology, Zhongshan Ophthalmic Center, Sun Yat-sen University, Guangzhou, 510060, China

E-mail: linxiaom@mail.sysu.edu.cn

**Table S1. Demographic characteristics of the participants with anisometropia amblyopia.**

| Subject | Gender | Age  (Y) | Amblyopia eye | Acuity (20/feet) | | Refraction | |
| --- | --- | --- | --- | --- | --- | --- | --- |
|  |  |  |  | Right Left | Left | Right | Left |
| AA01 | M | 18 | AE (L) | 20/12.5 | 20/62.55 | plano | +3.25DS/+2.25DC×80 |
| AA02 | M | 22 | AE (OU) | 20/100 | 20/100 | +10 DS/+0.5DC×180 | +11.5DS/+0.25DC×80 |
| AA03 | F | 18 | AE (R) | 20/200 | 20/12.5 | +6.5DS/+0.5DC×90 | plano |
| AA04 | F | 22 | AE (R) | 20/200 | 20/20 | -19.5DS/-1DC×65 | -6.0DS |
| AA05 | F | 18 | AE (R) | 20/80 | 20/32 | +8.0DS/+0.75DC×120 | +7.0DS/+1DC×60 |
| AA06 | F | 20 | AE (L) | 20/20 | 20/200 | -5.5DS | -20DS |
| AA07 | F | 17 | AE (R) | 20/50 | 20/20 | +2.75DS/-5.50DC×175 | +0.50DS/+0.50DC×90 |
| AA08 | F | 18 | AE (L) | 20/20 | 20/400 | -1.5DS/-0.25DC×85 | -13.0DS/-2.0DC×165 |
| AA09 | M | 17 | AE (L) | 20/20 | 20/32 | -2.5DS/-0.75DC×175 | -7.25DS/-0.50DC×180 |
| AA10 | M | 24 | AE (R) | 20/50 | 20/32 | +7.75DS | +6.0DS/+0.75DC×90 |
| AA11 | F | 30 | AE (L) | 20/16 | 20/50 | +0.50DS/+0.50DC×70 | +8.0DS/+1.0DC×120 |
| AA12 | M | 23 | AE (L) | 20/12.5 | 20/400 | +5.0DS | +7.0DS |
| AA13 | F | 43 | AE (L) | 20/20 | 20/50  20/20 | -0.5DC×75 | +3.0DS |

**Table S1(Continued).** Demographic characteristics of the participants with mixed amblyopia**.**

| Subject | Gender | Age  (Y) | Amblyopia eye | Acuity (20/feet) | | Refraction | |
| --- | --- | --- | --- | --- | --- | --- | --- |
|  |  |  |  | Right Left | Left | Right | Left |
| MA01 | M | 20 | AE (R) | 20/50 | 20/20 | +5DS/+2DC×90 | +3.5DS/+1DC×85 |
| MA02 | F | 28 | AE (L) | CF | 20/20 | -1.5DC×175 | -1.0DS |
| MA03 | F | 19 | AE (R) | 20/62.5 | 20/20 | -11DS/-3.5DC×175 | -6.25DS/-1DC×165 |
| MA04 | F | 21 | AE (L) | CF | 20/16 | +3.5DS | +6.0DS/+2DC×130 |
| MA05 | M | 20 | AE (R) | 20/50 | 20/50 | +5.0DS/+0.5DC×10 | -1.25DS |
| MA06 | M | 17 | AE (L) | 20/100 | 20/100 | +1.50DS/+0.50DC×100 | plano |
| MA07 | F | 27 | AE (L) | CF | 20/16 | +2.5DC×45 | plano |
| MA08 | M | 22 | AE (L) | 20/125 | 20/16 | -1.50DS/-2.5DC×160 | +0.25DS |
| MA09 | M | 17 | AE (R) | CF | 20/16 | +3.250DS/+1.25DC×80 | +1.25DS |
| MA10 | F | 20 | AE (L) | 20/200 | 20/12.5 | +4.0DS | -3.5DS/-0.5DC×90 |
| MA11 | F | 26 | AE (R) | 20/200 | 20/12.5 | +0.50DS/+1.0DC×50 | -1.25DS/-0.75DC×175 |
| MA12 | F | 19 | AE (L) | CF | 20/16 | +0.250DS/+1.0DC×10 | +0.50DS |
| MA13 | M | 20 | AE (L) | 20/200 | 20/200 | +4.50DS/+2.25DC×140 | +6.0DS |
| MA14 | M | 29 | AE (L) | 20/100 | 20/16 | +2.00DS/+2.25DC×140 | plano |
| MA15 | M | 45 | AE (R) | 20/40 | 20/16 | +1.0DC×65 | -0.25DS/-1.5DC×10 |

Table S2. Brain areas alterations in functional connectivity with the left primary visual area between anisometropic amblyopic subjects and normal sighted subjects (*P < 0.01*, 130 voxels, Alphasim corrected *P_alpha_* = 0.01)

| Brain Region | Brodmann  Region | Cluster  Size | T- scores | Z-scores | MNI Coordinates (x, y, z) |
| --- | --- | --- | --- | --- | --- |
| Cerebellum Crus 1/2 |  | 1977 | 5.39 | 4.47 | 36 -62 -30 |
|  |  |  | 5.27 | 4.40 | 18 -86 -36 |
|  |  |  | 4.81 | 4.11 | -20 -54 -48 |
| Cerebellum Crus1.L |  | 429 | 5.14 | 4.32 | -28 -56 -32 |
|  |  |  | 3.94 | 3.51 | -18 -74 -22 |
|  |  |  | 3.80 | 3.41 | -26 -72 -30 |
| Cerebellum Crus8/9.R |  | 207 | 4.57 | 3.95 | 20 -36 -48 |
|  |  |  | 3.75 | 3.37 | 18 -44 -48 |
|  |  |  | 3.43 | 3.12 | 32 -48 -54 |
| MFG/PreCG.L | 8/9 | 174 | 4.14 | 3.65 | -42 8 38 |
|  |  |  | 3.40 | 3.10 | -30 16 44 |
|  |  |  | 2.81 | 2.62 | -30 18 54 |
| IPL/ANG.L | 40 | 448 | 3.98 | 3.54 | -38 -58 44 |
|  |  |  | 3.55 | 3.22 | -38 -62 56 |
|  |  |  | 3.23 | 2.97 | -48 -66 50 |
| IPL/ANG.R | 40 | 263 | 3.61 | 3.27 | 40 -54 50 |
|  |  |  | 3.46 | 3.15 | 44 -62 54 |
|  |  |  | 2.88 | 2.68 | 48 -50 56 |
| SMG/ANG.R | 40 | 339 | 3.45 | 3.14 | 50 -52 24 |
|  |  |  | 3.37 | 3.08 | 56 -44 32 |
|  |  |  | 3.17 | 2.92 | 58 -42 48 |

Abbreviations: PostCG = postcentral gyrus, PreCG = precentral gyrus, MFG= middle frontal lobe, IPL = inferior parietal lobe, SMG = supramarginal, ANG = angular gyrus

L= left, R = right.

Table S3. Brain areas alterations in functional connectivity with the right primary visual area between anisometropic amblyopic subjects and normal sighted subjects (*P < 0.01*, 130 voxels, Alphasim corrected *P_alpha_* = 0.01)

| Brain Region | Brodmann  Region | Cluster  Size | T- scores | Z-scores | MNI Coordinates  (x, y, z) |
| --- | --- | --- | --- | --- | --- |
| Cerebellum Crus 1/2 / Lingual/Vermis_6/9 |  | 1174 | 4.80 | 4.10 | -26 -74 -30 |
|  |  |  | 4.73 | 4.06 | -8 -62 -20 |
|  |  |  | 4.50 | 3.90 | 8 -92 -26 |
| Cerebellum Crus 1/8/9.L |  | 236 | 4.36 | 3.80 | -36 -44 -40 |
|  |  |  | 4.21 | 3.70 | -22 -40 -52 |
|  |  |  | 3.79 | 3.40 | -24 -34 -46 |
| IPL/Angular.L | 40 | 300 | 3.80 | 3.41 | -40 -56 38 |
|  |  |  | 3.24 | 2.97 | -50 -66 44 |
|  |  |  | 3.21 | 2.95 | -38 -50 50 |
| Cerebellum Crus 1/6.R |  | 282 | 3.73 | 3.35 | 28 -60 -28 |
|  |  |  | 3.72 | 3.35 | 38 -64 -32 |
|  |  |  | 3.48 | 3.16 | 52 -66 -26 |
| PostCG.L | 2/3 | 327 | -4.34 | -3.79 | -68 -20 34 |
|  |  |  | -3.76 | -3.37 | -56 -12 18 |
|  |  |  | -3.69 | -3.32 | -46 -22 18 |
| PCL/MFG.L | 6/31 | 200 | -4.06 | -3.60 | -14 -30 58 |
|  |  |  | -3.69 | -3.33 | -10 -24 48 |
|  |  |  | -3.47 | -3.15 | -14 -32 48 |

Abbreviations: PostCG = postcentral gyrus, PCL=paracentral lobule, SMG = supramarginal

L= left, R = right.

Table S4 Brain areas alterations in functional connectivity with the left primary visual area between mixed amblyopic (anisometropic and strabismic) subjects and normal sighted subjects (*P < 0.01*, 130 voxels, Alphasim corrected *P_alpha_* = 0.01)

| Brain Region | Brodmann  Region | Cluster  Size | T-values | Z-scores | MNI Coordinates (x, y, z) |
| --- | --- | --- | --- | --- | --- |
| MFG | 11 | 211 | 5.70 | 4.70 | 6 58 -12 |
|  |  |  | 3.52 | 3.21 | -4 58 -18 |
|  |  |  | 3.01 | 2.80 | 16 52 2 |
| Cerebellum Crus 6/8/9 |  | 2586 | 5.18 | 4.38 | -10 -52 -54 |
|  |  |  | 4.34 | 3.82 | 38 -46 -36 |
|  |  |  | 4.19 | 3.71 | -28 -58 -32 |
| Cerebellum Crus 1 |  | 153 | 3.99 | 3.57 | -50 -72 -30 |
|  |  |  | 3.73 | 3.37 | -46 -80 -26 |
|  |  |  | 2.99 | 2.79 | -44 -68 -26 |
| Cerebellum Crus 6/Vermis_9 |  | 357 | 4.44 | 3.89 | 8 -54 -36 |
|  |  |  | 3.66 | 3.32 | -14 -48 -40 |
|  |  |  | 3.65 | 3.31 | 0 -50 -42 |
| PCC/PreCu | 30 | 178 | 5.17 | 4.37 | 2 -58 10 |
|  |  |  | 3.39 | 3.11 | -10 -62 16 |
|  |  |  | 2.92 | 2.73 | -6 -54 10 |
| IPL/ANG | 7/40 | 637 | 4.48 | 3.92 | 32 -56 46 |
|  |  |  | 4.18 | 3.70 | 32 -46 38 |
|  |  |  | 3.94 | 3.53 | 32 -62 54 |
| IPL/ANG.L | 7/40 | 988 | 4.09 | 3.64 | -38 -58 42 |
|  |  |  | 4.07 | 3.62 | -50 -42 38 |
|  |  |  | 4.05 | 3.61 | -38 -46 42 |
| ITG.L | 20 | 292 | 4.32 | 3.81 | -56 -16 -32 |
|  |  |  | 3.66 | 3.32 | -56 -38 -14 |
|  |  |  | 3.51 | 3.20 | -58 -46 -16 |
| Thalamus.L |  | 240 | 4.31 | 3.80 | -12 -18 14 |
|  |  |  | 3.10 | 2.88 | -10 -26 8 |
|  |  |  | 2.89 | 2.71 | -10 -8 6 |
| Thalamus.R |  | 199 | 3.41 | 3.13 | 14 -22 14 |
|  |  |  | 3.17 | 2.94 | 8 -6 0 |
|  |  |  | 3.15 | 2.92 | 8 -24 6 |

Abbreviations: MFC = medial frontal cortex, PostCG = postcentral gyrus, PCL=paracentral lobule, SMG = supramarginal, PreCu = precuneus, PCC = posterior cingulate cortex, IPL = inferior parietal lobule, ITG = inferior temporal, ANG = angular gyrus.

L= left, R = right.

Table S5. Brain areas alterations in functional connectivity with right primary visual area between mixed amblyopic (anisometropic and strabismic) subjects and normal sighted subjects (*P < 0.01*, 130 voxels, Alphasim corrected *P_alpha_* = 0.01)

| Brain Region | Brodmann  Region | Cluster  Size | T-scores | Z-scores | MNI Coordinates (x, y, z) |
| --- | --- | --- | --- | --- | --- |
| HIP/PHIP |  | 287 | 5.82 | 4.77 | -28 -30 -4 |
|  |  |  | 4.65 | 4.04 | -22 -22 -16 |
|  |  |  | 3.96 | 3.55 | -34 -20 -16 |
| PCC/MCC | 31 | 205 | 4.96 | 4.24 | -8 -40 40 |
|  |  |  | 3.19 | 2.95 | 4 -36 42 |
|  |  |  | 2.70 | 2.54 | -6 -36 32 |
| Lingual/Vermis_6 |  | 222 | 4.41 | 3.87 | -4 -82 -6 |
|  |  |  | 3.41 | 3.12 | 4 -74 -14 |
|  |  |  | 3.27 | 3.01 | -8 -88 -10 |
| PCC/PreCun.L |  | 293 | 4.08 | 3.63 | -20 -60 32 |
|  |  |  | 3.59 | 3.26 | -10 -56 10 |
|  |  |  | 3.37 | 3.09 | -20 -48 14 |
| Thalamus.L |  | 337 | 3.83 | 3.45 | 10 -22 6 |
|  |  |  | 3.54 | 3.23 | 2 -20 4 |
|  |  |  | 3.46 | 3.17 | -8 -14 14 |
| IPL/ANG.L | 40 | 204 | 3.77 | 3.41 | -32 -56 40 |
|  |  |  | 3.23 | 2.98 | -38 -58 32 |
| PreCu | 31 | 228 | 3.77 | 3.40 | 18 -56 26 |
|  |  |  | 3.44 | 3.15 | 10 -68 26 |
| PostCG/PreCG.L | 3/4 | 142 | 4.28 | 3.78 | -62 -14 44 |
|  |  |  | 2.72 | 2.56 | -60 -18 34 |
|  |  |  | 2.64 | 2.49 | -52 -24 60 |
| MOG | 19 | 137 | 4.08 | 3.64 | 48 -74 -2 |
|  |  |  | 3.14 | 2.91 | 40 -84 -2 |
|  |  |  | 2.62 | 2.47 | 46 -78 8 |
| PostCG/PreCG.R | 3/4 | 175 | 3.72 | 3.36 | 52 -28 50 |
|  |  |  | 3.52 | 3.22 | 60 -20 34 |
|  |  |  | 3.09 | 2.87 | 54 -20 58 |
|  |  |  | 3.09 | 2.87 | 54 -20 58 |

Abbreviations: PostCG = postcentral gyrus, PreCG = precentral gyrus, PCL= paracentral lobule, SMG = supramarginal, PreCu = precuneus, PCC = posterior cingulate cortex, IPL = inferior Parietal Lobule, ITG = inferior temporal gyrus, ANG = angular gyrus, MOG = middle occipital cortex. HIP/PHIP = hippocampus/parahippocampus.

L= left, R = right.
